# Novel Debris Material Identification Method Based on Impedance Microsensor

**DOI:** 10.3390/mi16070812

**Published:** 2025-07-14

**Authors:** Haotian Shi, Yucai Xie, Hongpeng Zhang

**Affiliations:** 1 College of Safety Science and Engineering, Nanjing University of Science and Technology, Nanjing 210094, China; dmu6hao@163.com; 2 Marine Engineering College, Dalian Maritime University, Dalian 116026, China

**Keywords:** oil condition monitoring, impedance debris sensor, material identification, coil-based impedance detection

## Abstract

Oil condition monitoring can ensure the safe operation of mechanical equipment. Metal debris is full of friction information, and the identification of debris material helps to locate wear of parts. A method based on impedance analysis is proposed to identify debris material in this article. The differences in permeability and conductivity result in the nonlinear variation trend of inductance–resistance amplitude with debris volume. By establishing a database of amplitude–size curves, debris information (material and size) can be obtained through impedance analysis. Based on experimental and simulation results, iron, stainless steel, aluminum, copper, and brass particles are effectively distinguished. This method is not affected by oil’s light transmittance, other impurities, and debris surface dirt and can be used to distinguish metals with similar colors. This work provides a novel solution for debris material identification, which is expected to promote the development of fault diagnosis.

## 1. Introduction

Currently, mechanical equipment is developing toward high-precision, high-efficiency, and intelligence; thus, its reliability requirements are higher. As an advanced technology for ensuring the safety of mechanical equipment, condition monitoring and fault early warning under big data are developing rapidly. In addition to the external environment and human factors, system failures mainly stem from abnormal coordination caused by wear [1,2]. Condition monitoring technology mainly includes vibration monitoring, oil analysis, sound feedback, and thermal imaging. Vibration monitoring, sound feedback, and thermal imaging are less sensitive to the health conditions of mechanical systems due to environmental noise and temperature hysteresis. It is difficult to find abnormal wear in the early stage [3,4]. Lubrication systems can inhibit wear, and debris generated by mechanical friction and external intrusions is carried in lubricating oil. Oil condition monitoring can identify and predict mechanical faults [5]. By detecting and analyzing the contaminants in oil, the lubrication environment and wear condition of parts can be directly reflected.

Oil condition monitoring can be divided into oil property analysis and oil contamination monitoring [6]. Oil property analysis reveals its lubricating performance through the degree of oil deterioration. Oil contamination monitoring focuses on measuring different content to reflect wear trends, such as water, air, and debris [7]. In particular, metal debris is full of friction information, which can be used as an indicator of mechanical health. The size and concentration of metal debris represent the wear level, while the wear mechanism can be judged based on debris morphology. Therefore, oil debris monitoring (ODM) can provide important information for intelligent operation. ODM includes offline, inline, online, and onsite forms. Offline detection in the laboratory can obtain accurate, detailed, and comprehensive information. However, due to the sample delivery link, offline detection results lag, which cannot meet the requirements of condition-based maintenance. Real-time information enables operators to actively respond to sudden failures and take emergency remedial measures. Therefore, the current demand for inline, online, and onsite oil detection devices with stronger timeliness is more urgent. The core components of these devices are sensors based on various principles, mainly including optics [8,9], acoustics [10,11], impedance [12,13,14], and image methods [15,16].

Metal materials of mechanical parts are different, and the debris material helps to semi-qualitatively determine the wear location. Material identification has always been a critical problem in ODM. At present, the distinction of debris material is mainly based on spectral and image recognition methods [17,18], but their detection effectiveness is greatly affected by oil’s light transmittance. It is of great significance to develop new debris material identification methods to provide more detailed information for fault diagnosis. It is worth noting that inductance methods based on electromagnetic induction can distinguish between ferromagnetic debris and non-ferromagnetic debris [19,20]. Recently, various inductance debris detection devices were studied and developed, mainly including self-inductance single-coil sensors [21], mutual-inductance double-coil sensors [22], balanced triple-coil sensors [23], etc. But material identification is also a weakness in inductive debris monitoring [24]. The metal debris information obtained by inductance detection methods needs to be extended from magnetic property differentiation to material identification. Our group has conducted research on debris material identification based on inductive sensors. Shi et al. [25] achieved metal debris distinction based on the differences in sensing characteristics between sensing units and analyzed the influence of coil turns and magnetic media on sensitivity. However, the sensor structure mentioned above is complex, and the distinguishing accuracy of debris materials still needs to be improved.

The electromagnetic field generated by metal debris will change the impedance parameters of the coil. Traditional inductive sensors present the impedance change in the form of voltage, ignoring that impedance is the combined result of inductance and alternating current (AC) resistance. The material information of the debris is contained in impedance signals. Permeability and conductivity depend on the metal material, and the characteristic curves between signal amplitude and particle size for various metal particles have different trends. By combining the debris information judged by inductance and resistance amplitude–size curves, the material and size can be determined. Therefore, this article proposes a debris material identification method based on impedance analysis to enrich the obtained debris information. And this method is not affected by oil’s light transmittance, other impurities, and debris surface dirt.

## 2. The Coil-Based Impedance Detection Method

### 2.1. The Impedance Debris Microsensor

**Figure 1:**
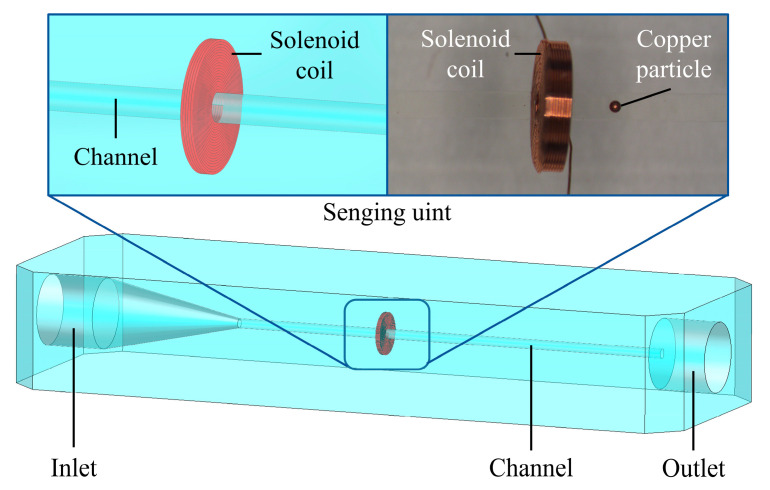
The impedance debris microsensor with Coulter Counting.

In this work, an impedance debris microsensor with Coulter Counting was selected, as shown in Figure 1. The sensor includes a sensor substrate, an oil inlet, an oil outlet, a solenoid coil, and a channel with a diameter of 900 μm. The coil parameters are as follows: 200 turns, 0.9 mm inner diameter, 3.7 mm outer diameter, and 0.7 mm height. The channel is perpendicular to the coil inner hole.

### 2.2. The Impedance Detection Principle Based on Coil

**Figure 2:**
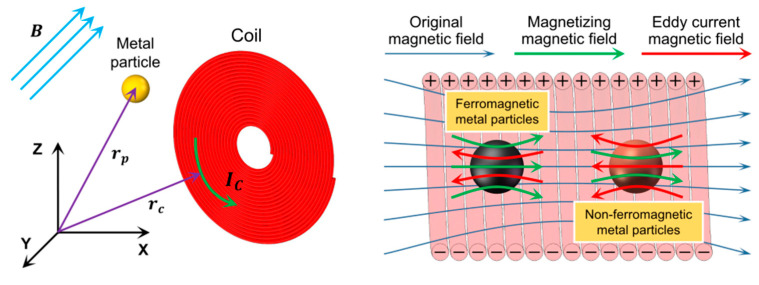
Detection principle based on alternating magnetic field.

With the magnetization effect enhancing and eddy current weakening the magnetic field, the metal debris will change the magnetic flux and break the balance of the skin effect and the proximity effect, thereby causing changes in coil inductance and AC resistance, as shown in Figure 2. By measuring the impedance change of the coil, metal debris can be identified, sized, and counted.

Based on previous research [26], the impedance change $Z_{m}$ caused by a metal particle in the self-excited coil is given by:

(1) $$Z_{m}\left(r_{m}\right)=j\mu_{v}V\chi_{m}{\left|h\left(r_{m}\right)\right|}^{2}$$

where $r_{m}$ is the position vector of the particle center, and $\mu_{v}$ is the permeability of vacuum, V is the volume of the metal particle. h is the sensitivity field of the coil:

(2) $$h\left(r_{m}\right)=\frac{1}{4\pi }\underset{coil}{∭}\frac{ndl\times \left(r_{R}-r_{E}\right)}{{\left|r_{R}-r_{E}\right|}^{3}}ds$$

where n is the turn density of the coil, dl is the current direction of the coil element, $r_{R}$ is the position vector of the receiver current element, and $r_{E}$ is the position vector of the excitation current element. $\chi_{m}$ is the magnetic susceptibility of the metal particle:

(3) $$\chi_{m}=\frac{3}{2}\frac{\left(2\mu_{m}+1-a^{2}k^{2}\right)\sin ak+ak\left(2\mu_{m}+1\right)\cos ak}{\left(\mu_{m}-1+a^{2}k^{2}\right)\sin ak-ak\left(\mu_{m}-1\right)\cos ak}$$

where $\mu_{m}$ is the relative permeability of the metal, a is the radius of the metal particle. k is given by:

(4) $$k=\sqrt{-j\omega \mu_{v}\mu_{m}\sigma_{m}}$$

Here, ω is the angular frequency of the alternating magnetic field and $\sigma_{m}$ is the conductivity of the metal.

The impedance change of coil includes inductance and AC resistance:

(5) $$L_{m}\left(r_{m}\right)=\mu_{v}V{\left|h\left(r_{m}\right)\right|}^{2}\mathrm{Real}\left(\chi_{m}\right)$$

(6) $$R_{m}\left(r_{m}\right)=-\mu_{v}V{\left|h\left(r_{m}\right)\right|}^{2}\mathrm{Imaginary}\left(\chi_{m}\right)$$

As concluded from the above equations, it can be found that the impedance signal mainly depends on the sensor structure (coil parameters and channel position), excitation parameters (voltage and frequency), and metal debris (volume and material). The material represents magnetic permeability and conductivity, which affect the intensity of magnetization and the eddy current. This provides a theoretical basis for the material identification of metal debris.

### 2.3. The Simulation Analysis of Impedance Detection

**Figure 3:**
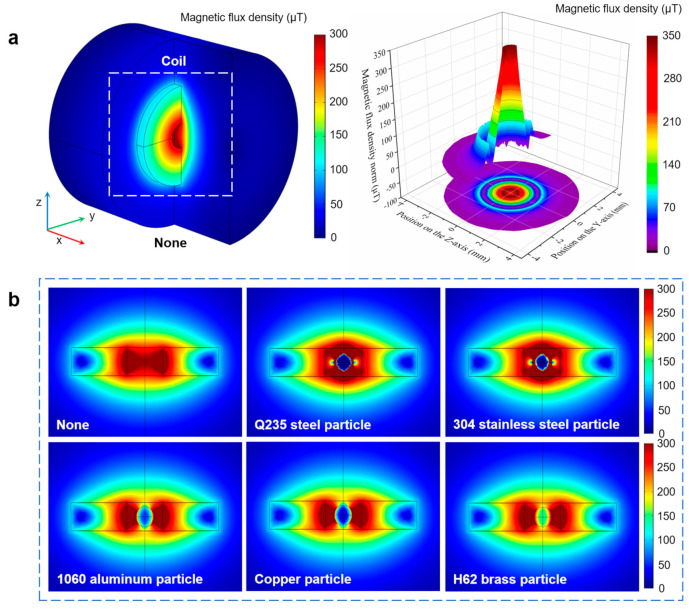
Simulation result of impedance sensor by COMSOL 5.4 (2.0 V, 2.0 MHz). (**a**) The distribution cloud diagram of the magnetic field around the coil. (**b**) The characteristics of magnetization and eddy current in various metal particles.

To verify the theoretical analysis, simulations of impedance detection were conducted. As shown in Figure 3, a simulation model was built according to the coil structure used in the experiment, and the characteristics of magnetization and the eddy current with 500 μm metal particles were obtained. After applying a 2.0 V, 2.0 MHz excitation current, the distribution cloud diagram of the magnetic flux density originating from the coil is shown in Figure 3a. The magnetic flux density distributed in the coil inner hole ranged between 300 μT (inner hole center) and 350 μT (inner hole edge). The magnetic gradient established by the micro-coil was small, which reduced the signal fluctuation induced by the particle position. This was conducive to improving the detection stability.

**Figure 4:**
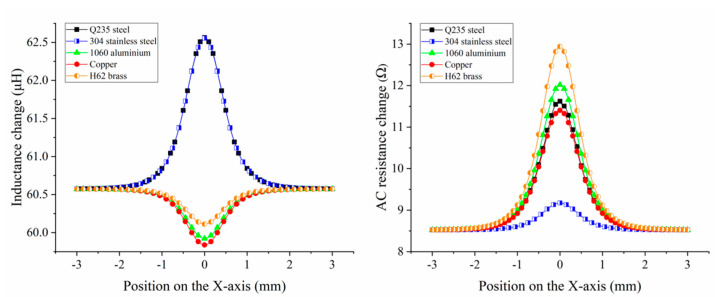
Inductance and AC resistance changes caused by 500 μm particles of different materials.

In engineering, the materials of various friction pairs are different. The debris material helps to achieve wear localization. This article selected several typical metals for research, such as iron, stainless steel, aluminum, copper, brass, etc. The simulation results for a 500 μm Q235 steel particle, 304 stainless steel particle, 1060 aluminum particle, copper particle, and H62 brass particle are shown in Figure 3b. The magnetization field and eddy current field produced by the metal particles cancelled each other. Owing to the differences in permeability and conductivity, the ratio of magnetization to the eddy current of metal debris was nonidentical. For ferromagnetic particles, the magnetization field was dominant, which would enhance the inductance. For non-ferromagnetic particles, the eddy current field was dominant, which would weaken the inductance. Under the combined action of the two fields, AC resistance would increase. The magnetic permeability of the Q235 steel particle was higher, thus its magnetization effect was stronger than that of the 304 stainless steel particle. That is, $\sigma_{m}$ of copper > $\sigma_{m}$ of 1060 aluminum > $\sigma_{m}$ of H62 brass, which made the eddy current in the copper particle the most intense, while the eddy current in the H62 brass particle was the weakest. The magnetization and eddy current characteristics could not intuitively reflect the changes in coil inductance and resistance. Therefore, the impedance signals caused by metal particles were obtained through parameterized scanning, as shown in Figure 4. The simulation data indicated that the inductance–resistance signal characteristics of various metals were different. The inductance pulses from the Q235 steel particle and 304 stainless steel were extremely close (the pulse amplitude of Q235 steel was 7.14 nH larger than that of 304 stainless steel), while the difference in resistance pulses was obvious (the pulse amplitude of Q235 steel was 2.449 Ω larger than that of 304 stainless steel). In addition, there were significant differences in the inductance and resistance pulses between the selected non-ferromagnetic metal particles. The amplitude–size curves for various metal particles had different trends. This was consistent with the theoretical analysis. Therefore, we propose a debris material identification method based on impedance analysis.

## 3. Experiments and Discussion

To verify the feasibility of the coil-based impedance detection method to distinguish debris material, the debris microsensor was fabricated based on the casting method [22]. The fabricating process included the following steps: coil winding, basic mold making, substrate curing, and channel forming. An Agilent E4980A meter (Agilent Technologies Inc., Bayan Lepas, Malaysia) was used to provide a 2.0 V, 2.0 MHz excitation for the sensor and to detect the impedance parameters. The 500 μm metal particle was added from the inlet and driven into the channel by a LSP02-1B syringe pump (Keysight E4980A, Baoding Qili Constant-flow-pump Ltd., Baoding, China). The high-frequency noise in the impedance signal was filtered out through a low-pass filter, and interpolation fitting method was used to achieve baseline zeroing. The measured data were processed to obtain the type, size, and number of debris.

**Figure 5:**
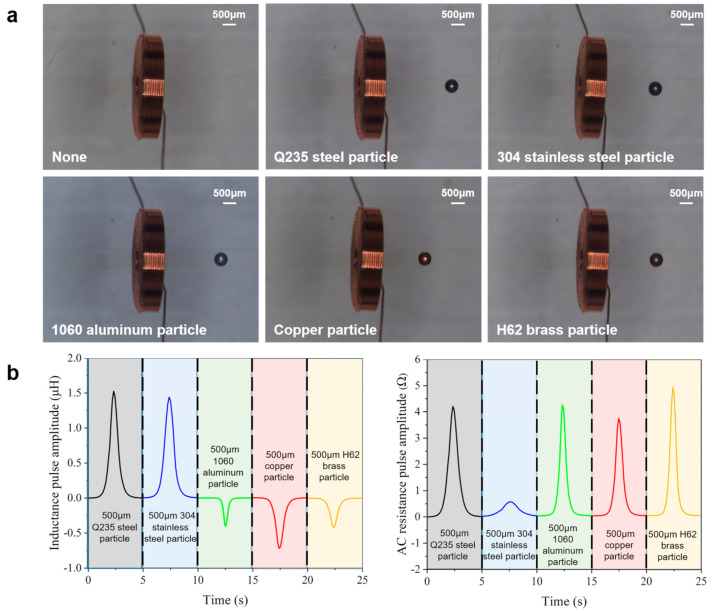
Research on the coil-based impedance detection method for identifying debris material. (**a**) The photographs of metal particles under the microscope. (**b**) The detection signals of 500 μm metal particles.

The Q235 steel particle, 304 stainless steel particle, 1060 aluminum particle, copper particle, and H62 brass particle were repeatedly driven through the sensing region for detection. The photographs of metal particles under the microscope are shown in Figure 5a. The detection signals of 500 μm metal particles of different materials are shown in Figure 5b. Q235 steel and 304 stainless steel are paramagnetic materials, which produced positive inductance pulses. 1060 aluminum, copper, and H62 brass are diamagnetic materials, which produced negative inductance pulses. And all metal particles produced positive resistance pulses. Compared with the simulation results, there were errors caused by sensor manufacturing, circuit loss, and metal particles themselves. For instance, the 500 μm 1060 aluminum particle produced a −0.403 μH inductance pulse and a 4.241 Ω resistance pulse, which was less than the simulation result. The error source was that the aluminum particle was affected by oxidation, and the effective size was small. Therefore, for metal debris with obvious oxidation characteristic, the oxidation volume should be considered in actual size judgment.

**Figure 6:**
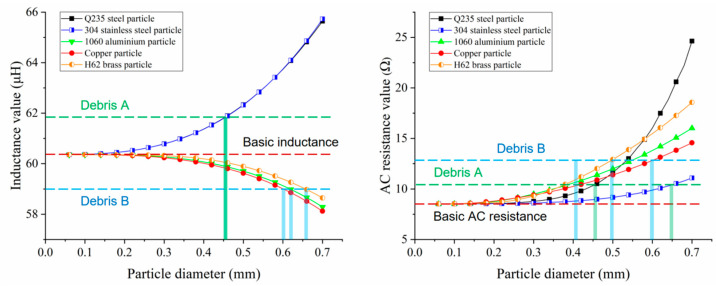
The characteristic curves of the inductance–resistance signals for various metals.

The coil-based impedance detection method was used to identify debris material by database comparison. The characteristic curves of the inductance–resistance signals for various metals were obtained in simulations, as shown in Figure 6. The results showed that the inductance–resistance pulse amplitudes grew nonlinearly with particle diameter. Eddy current and magnetization effects increased sharply with the particle volume, and the disturbances to the coil magnetic flux and the current distribution in the wire were more significant. With the detected impedance signal, metal debris could be analyzed according to the amplitude–size curves. A single inductance pulse amplitude or resistance pulse amplitude corresponded to multiple metal debris. By combining the debris information judged by inductance and resistance amplitude–size curves, the material and size could be determined. For example, debris A generated a 1.50 μH inductance pulse and a 2.00 Ω resistance pulse, while debris B generated a −1.50 μH inductance pulse and a 4.65 Ω resistance pulse. Selecting the simulation characteristic curves as the standard database, the debris information is analyzed in Figure 6. According to the direction (positive or negative) of the inductance pulses, particles could be determined to be ferromagnetic or non-ferromagnetic. Based on the inductance amplitude–size curves, debris A was judged as a 455 μm Q235 steel particle or 460 μm 304 stainless steel particle, and debris B was judged as a 620 μm copper particle, 640 μm 1060 aluminum particle, or 670 μm H62 brass particle. Based on the resistance amplitude–size curves, debris A was judged as a 455 μm Q235 steel particle or 660 μm 304 stainless steel particle, and debris B was judged as a 620 μm copper particle, 560 μm 1060 aluminum particle, or 510 μm H62 brass particle. Based on the comprehensive analysis of the two results, debris A was identified as 455 μm Q235 steel, and debris B was identified as 620 μm copper.

This debris material identification method relies on the predefined database of amplitude–size curves. In practical applications, particles that cannot be identified by a predefined database will be classified as unknown particles. Based on the inductance changes, these particles can be distinguished as ferromagnetic and non-ferromagnetic, and their size can be roughly estimated.

The debris material identification method based on impedance analysis is not affected by oil’s light transmittance, other impurities, and debris surface dirt. And this method can be used to distinguish metals with similar colors. The greater difference between the amplitude–size curves, the more accurate the identified information. Therefore, the identification accuracy of debris material can be elevated by enhancing sensor performance. Furthermore, the pulse of small metal particles will be submerged in signal noise. The research and development of high-sensitivity impedance debris sensors is of great significance for the collection of debris information. In addition, this method can be combined with another method proposed in previous work [25], and impedance signal analysis based on multi-sensing units can improve the accuracy of debris material identification.

## 4. Conclusions

In conclusion, a coil-based impedance detection method is proposed to identify debris material. Firstly, the theoretical foundation of this method is established. The magnetization and eddy current differences between various particles are proven by electromagnetic simulations. The impedance microsensor fabricated using the casting method is used to study the inductance–resistance signal characteristics of metal debris. As each material has its unique permeability and conductivity, the nonlinear change trend of inductance and resistance pulse amplitude with debris volume is illustrated. Through database comparison, the method can effectively identify debris material. The identification accuracy of debris material can be improved by increasing the difference between the amplitude–size curves. This work provides a novel solution for debris material identification, enriching the detection information of debris sensors for mechanical fault diagnosis.

## Data Availability

Dataset available on request from the authors.
